# *B3GALNT2* mutations associated with non-syndromic autosomal recessive intellectual disability reveal a lack of genotype–phenotype associations in the muscular dystrophy-dystroglycanopathies

**DOI:** 10.1186/s13073-017-0505-2

**Published:** 2017-12-22

**Authors:** Reza Maroofian, Moniek Riemersma, Lucas T. Jae, Narges Zhianabed, Marjolein H. Willemsen, Willemijn M. Wissink-Lindhout, Michèl A. Willemsen, Arjan P. M. de Brouwer, Mohammad Yahya Vahidi Mehrjardi, Mahmoud Reza Ashrafi, Benno Kusters, Tjitske Kleefstra, Yalda Jamshidi, Mojila Nasseri, Rolph Pfundt, Thijn R. Brummelkamp, Mohammad Reza Abbaszadegan, Dirk J. Lefeber, Hans van Bokhoven

**Affiliations:** 1 Genetics and Molecular Cell Sciences Research Centre, St George’s University of London, Cranmer Terrace, London, SW17 0RE UK; 2 Department of Neurology, Radboud university medical center, Geert Grooteplein 10, 6525 GA Nijmegen, The Netherlands; 3 Department of Laboratory Medicine, Radboud university medical center, Geert Grooteplein 10, 6525 GA Nijmegen, The Netherlands; 4 Department of Human Genetics 855, Donders Institute for Brain, Cognition and Behaviour, Radboud university medical center, Geert Grooteplein 10, 6525 GA Nijmegen, The Netherlands; 5 Gene Center and Department of Biochemistry, Ludwig-Maximilians-Universität München, Feodor-Lynen-Straße 25, 81377 Munich, Germany; 6 Pardis Clinical and Genetics Laboratory, Mashhad, Iran; 7 Medical Genetics Research Center, Shahid Sadoughi University of Medical Sciences, Yazd, Iran; 8 Department of Child Neurology, Children’s Medical Center, Tehran University of Medical Sciences, Tehran, Iran; 9 Department of Pathology, Radboud university medical center, Geert Grooteplein 10, 6525 GA Nijmegen, The Netherlands; 10 Department of Pathology, Maastricht University Medical Centre, 6229 HX Maastricht, The Netherlands; 11 Medical Genetics Research Center, Mashhad University of Medical Sciences, Mashhad, Iran; 12 Division of Human Genetics, Immunology Research Center, Avicenna Research Institute, Mashhad University of Medical Sciences, Mashhad, Iran

**Keywords:** Dystroglycan, *B3GALNT2*, Muscular dystrophy-dystroglycanopathy syndrome, Intellectual disability, Epilepsy

## Abstract

**Background:**

The phenotypic severity of congenital muscular dystrophy-dystroglycanopathy (MDDG) syndromes associated with aberrant glycosylation of α-dystroglycan ranges from the severe Walker-Warburg syndrome or muscle-eye-brain disease to mild, late-onset, isolated limb-girdle muscular dystrophy without neural involvement. However, muscular dystrophy is invariably found across the spectrum of MDDG patients.

**Methods:**

Using linkage mapping and whole-exome sequencing in two families with an unexplained neurodevelopmental disorder, we have identified homozygous and compound heterozygous mutations in *B3GALNT2*.

**Results:**

The first family comprises two brothers of Dutch non-consanguineous parents presenting with mild ID and behavioral problems. Immunohistochemical analysis of muscle biopsy revealed no significant aberrations, in line with the absence of a muscular phenotype in the affected siblings. The second family includes five affected individuals from an Iranian consanguineous kindred with mild-to-moderate intellectual disability (ID) and epilepsy without any notable neuroimaging, muscle, or eye abnormalities. Complementation assays of the compound heterozygous mutations identified in the two brothers had a comparable effect on the O-glycosylation of α-dystroglycan as previously reported mutations that are associated with severe muscular phenotypes.

**Conclusions:**

In conclusion, we show that mutations in *B3GALNT2* can give rise to a novel MDDG syndrome presentation, characterized by ID associated variably with seizure, but without any apparent muscular involvement. Importantly, *B3GALNT2* activity does not fully correlate with the severity of the phenotype as assessed by the complementation assay.

## Background

Congenital muscular dystrophy-dystroglycanopathy (MDDG) syndromes are a group of inherited disorders with a broad clinical phenotype [1]. The most severe form is Walker-Warburg syndrome (WWS), which has an onset at birth and is characterized by severe muscular dystrophy, cobblestone lissencephaly, cerebellar abnormalities, hydrocephalus, and eye malformations. Patients with muscle-eye-brain disease (MEB) and Fukuyama congenital muscular dystrophy (FCMD) have a similar but less severe phenotype. The mildest form of MDDG syndrome is limb-girdle muscular dystrophy (LGMD), which has a later age of onset and in which only a small proportion of patients present with mild structural brain abnormalities [2, 3] or dilated cardiomyopathy [4]. However, muscular dystrophy is the hallmark of all MDDG syndromes described so far.

MDDG syndromes are caused by defective O-glycosylation of α-dystroglycan (α-DG), leading to reduced α-DG-laminin binding in the extracellular matrix [5, 6]. Mutations in *DAG1* encoding α-DG and β-DG, and in 17 other genes, encoding proteins involved in the O-glycosylation pathway, cause different forms of MDDG syndrome [7–9].

Thus far, ten patients with mutations in the *B3GALNT2* (NM_152490.4) have been described [10–12]. These patients have various combinations of missense and truncating mutations associated with variable but consistently severe phenotypes and with congenital muscular dystrophy in all cases. In this work, we describe two families with seven affected individuals that present with a novel atypical and very mild form of MDDG resulting from compound heterozygous and homozygous mutations, respectively, in *B3GALNT2*. In addition, complementation assays were performed to analyze the effect of the various mutations on the O-glycosylation of α-DG.

## Methods

### Patients

The patients from family A were ascertained in a large cohort study comprising individuals with unexplained intellectual disability (ID) at the Department of Human Genetics of the Radboud University Medical Center in Nijmegen, the Netherlands, where they had a thorough genetic diagnostic evaluation [13, 14]. Members of family B were recruited through Pardis Genetic laboratory in Mashhad, Iran. The parents consented to this study and the study was approved by the local ethical committees.

### Genetic study

DNA was extracted from peripheral blood, using standard procedures. For Family A, exome sequencing was carried out for both the affected brothers. Exome enrichment was performed using the SureSelectXT Human All Exon 50 Mb Kit (Agilent, Santa Clara, CA, USA). DNA samples were multiplexed and sequenced using the SOLiDTM 4 System, leading to 6.09 Gb and 7.22 Gb of mappable sequence, respectively. Read mapping and variant calling was performed with SOLiD bioscope software v1.3 using hg19 as the human reference genome. For Family B, a genome-wide single nucleotide polymorphism (SNP) genotyping analysis was first undertaken in four affected (IV:1, IV:3, IV:6, V:2) and two unaffected individuals (IV:7; IV:9) using the Illumina HumanCytoSNP-12 v2.1 chip array (330 K markers) for autozygosity mapping. This was followed by whole exome sequencing (WES) of genomic DNA from proband (V:2) performed at Otogenetics Corporation (Norcross, GA, USA) using the Agilent SureSelect Human All ExonV4 (51 Mb) enrichment kit with a paired-end (2 × 100) protocol at a mean coverage of 30X. Reads were aligned to genome assembly hg19 with the Burrows-Wheeler Aligner (BWA, V.0.5.87.5).

### Biochemical analysis

Staining for α-DG (IIH6; mouse monoclonal IgG antibody, 1:1000, Millipore) was carried out on patient skeletal muscle sections as described previously [15].

### Cell culture

Human haploid HAP1 cells [16] were cultured in Iscove’s modified Dulbecco’s medium (IMDM, Gibco) supplemented with 10% fetal bovine serum and 1% Penicillin/streptomycin/L-glutamine (Gibco) at 37 °C under 5% CO_2_ atmosphere.

### Complementation of *B3GALNT2*-deficient HAP1 cells

*B3GALNT2* complementary DNA (cDNA) was cloned into a retroviral expression vector, pBabe-puro, using EcoRI and SalI restriction sites as previously described [17]. Mutant constructs were obtained by site-directed-mutagenesis using Phusion® High Fidelity DNA Polymerase (New England Biolabs), Q-solution (Qiagen), and five pairs of primers (primer sequences available upon request): viruses expressing wild-type (WT) and mutant *B3GALNT2* were produced in 293 T cells and used to infect HAP1 *B3GALNT2*-deficient cells as described previously [10].

### Flow cytometry analysis

HAP1 WT, *B3GALNT2*-deficient, and complemented cells were incubated with IIH6-C4 antibody (Millipore), followed by incubation with goat anti-mouse Alexa Fluor568 antibody (Invitrogen). Subsequently, the fluorescence signal was measured at a BD Fortessa flow cytometer as described previously [17].

## Results

### Patient phenotype

#### Family A

**Fig. 1:**
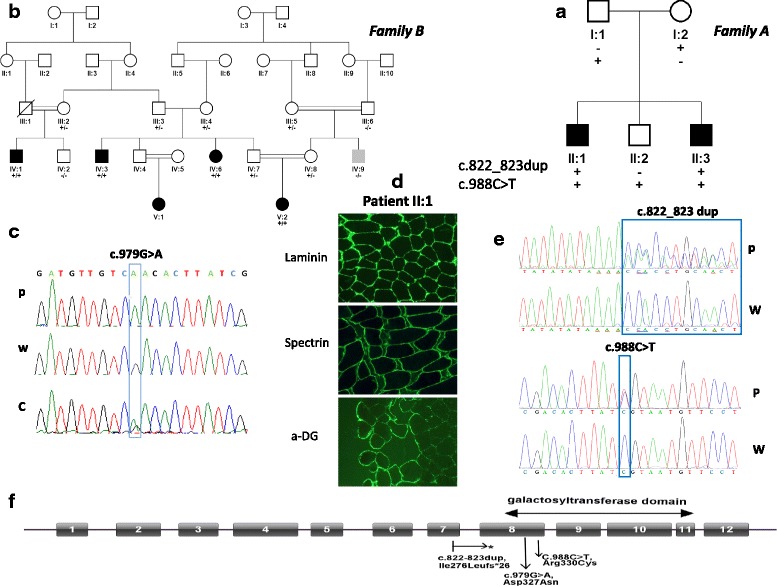
Genetic and biochemical analysis. **a**, **b** Pedigrees of the two families showing segregation of the mutated alleles within the families. The affected individuals are shown as *black symbols* and the *gray symbol* shows a 28-year-old individual with only borderline learning difficulty and attention deficit hyperactivity disorder without epilepsy. Mutant alleles shown by “+” and WT allele shown by “−.” **c**, **e** Sections of Sanger sequencing chromatograms for the mutations, heterozygous, and WT alleles are depicted and location of the alteration is demonstrated in the box. W wildtype, C carrier, P patient. **d** Immunohistochemistry of skeletal muscle of patient II-1 showed a minimal reduction of α-DG staining compared to a healthy control. α-DG staining was performed using the IIH6 antibody, recognizing the laminin-binding glycol-epitope. Spectrin and laminin staining were performed as control. **f** Schematic overview of the *B3GALNT2* structure and the mutations identified in both families. The duplication leads to a premature stop codon (*), resulting in a truncated transcript lacking the galactosyltransferase domain. The missense mutations are located on exon 8 and cause substitution of a strongly conserved residue within the galactosyltransferase domain

**Fig. 2:**
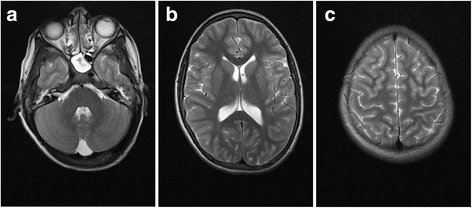
T2-weighted MRI of the brain of patient II-1 at the age of 12 years. The *images* illustrate the normal appearance of the cerebellum and the pons (**a**) and the normal signal intensities of the cerebellar and cerebral white matter as well as the normal development of the cerebral cortex (**b**, **c**)

Patients II-1 and II-3, from a Dutch non-consanguineous family, are two affected male siblings aged 14 and 8 years at presentation. They have a healthy brother (Fig. 1a). Patient II-1 was born after an uncomplicated pregnancy and birth, with a normal birth weight of 3655 g (50th–75th centile). He was a very quiet baby. His psychomotor development was delayed. Speech development was delayed more than motor development. He has walked independently since the age of 21 months. He started to speak his first words at the age of three years. At the age of 11 years he still had trouble telling simple stories and was diagnosed with dysphasia. He could read at beginners’ level. A formal intelligence test yielded an IQ of 55. His behavior was characterized by temper tantrums and features of autism spectrum disorder. Hearing and vision were normal. At the age of 10 years 11 months, he had a normal height (143 cm/20th centile) and weight (34 kg/50th centile) and a low normal head circumference (52 cm/5th centile). There were no facial dysmorphic features observed. Neurological evaluation revealed dysphasia and he had symmetrical low tendon reflexes, but no further signs of pyramidal, extrapyramidal, cerebellar, or neuromuscular problems were observed. Brain magnetic resonance imaging (MRI) at the age of three years showed mild bilateral periventricular white matter signal abnormalities. At the age of 12 years, brain MRI was repeated and found to be normal (Fig. 2). The creatine kinase (CK) level was slightly elevated (187 U/L; normal < 170 U/L). A muscle biopsy including spectrin, laminin, and glycosylated α-DG staining showed an intact muscle structure and no significantly abnormal α-DG staining (Fig. 1b). Genome-wide chromosomal analysis by 250 K SNP array analysis and a metabolic screen revealed no abnormalities.

The younger brother, patient II-3, was born after an uncomplicated pregnancy and birth. He had a normal birth weight of 3495 g (50th centile). His psychomotor development was delayed with independent walking after the age of two years and a severe speech delay with only five single words at the age of three years. At the age of 5 years 4 months, his speech was only intelligible to his parents and his articulation was poor. A formal intelligence test showed a total IQ of 68. He had hyperactive behavior and a need for structure, but better social interaction than his brother, though contact was mainly functional. He occasionally displayed aggressive behavior and had problems falling asleep. His attention span was very short. Hearing and vision were normal. At the age of 5 years 4 months, he had a normal height (114.5 cm/50th centile), weight (20 kg/50th centile), and head circumference (51.4 cm/50th centile). He had no evident facial dysmorphic features. Upon clinical neurological evaluation intelligibility was poor, but his speech was not truly dysarthric. He had no signs of pyramidal, extrapyramidal, cerebellar, or neuromuscular dysfunctioning. As with his brother, genome-wide chromosomal analysis by 250 K SNP array analysis revealed no abnormalities. Therefore, both were included in exome sequencing studies assuming an autosomal recessive or X-linked inheritance pattern.

#### Family B

This is a large, consanguineous Iranian kindred with five individuals, three male and two female, aged 3–37 years across four branches, affected by unexplained autosomal recessive developmental delay (DD), ID, and epilepsy. Similar to family A, speech is more severely affected than motor development in the affected members of the family. All the patients were born after normal pregnancies from healthy parents who are related. Karyotype analysis by G-banding and tandem mass spectrometry screening for metabolic disorders as well as Fragile X screening were performed for all affected individuals and they were normal. Results of brain computed tomography (CT) scans taken for all the patients were unremarkable. The results of routine laboratory testing and CK level was within normal range. Physical examination, dysmorphology examination, and neurological assessment did not find anything unusual except for cognition deficiency and history of seizure. The patients did not have any neurological problems, autistic features, behavioral abnormalities, nor congenital malformations. Growth parameters including height, weight, head circumference, and general health were normal and there were no clinical or biochemical indications of other systems involvement. Hearing and ophthalmologic (retina and optic nerve) examinations did not find any defect. None of the patients had any signs of muscle weakness or muscle atrophy.

The proband (V:2) of the family is a nine-year-old girl who was born by a Cesarean-section delivery. She was cyanotic at birth but otherwise normal. She had a history of seizures starting at the age of 15 days and was under treatment with Phenobarbital between the ages of 4 and 8 months. Her EEG result was abnormal at the time, but she is currently seizure-free. She had psychomotor delay and with physiotherapy she started to walk at the age of two years. She also had speech delay (only three-word sentences). After speech therapy, her speech has improved significantly and she speaks much better although it is still not comprehensible. She has mild to moderate ID. Her formal intelligence test yielded an IQ of < 50 with need of special education schooling. She is usually a quiet girl and no behavioral abnormalities were noticed. Brain MRI at the age of 18 months showed high signal intensity along the periventricular white matter and leukodystrophy was suggested as a possible diagnosis. However, at the age of seven years, brain MRI was repeated and found to be normal.

Individuals IV:3 and IV:6 are two brothers aged 34 year and 41 years, respectively, who are maternal uncles of the proband with mild ID (IQ:50) and epileptic seizures until the age of five years. Both brothers remain illiterate past adolescence. However, they are able to take care of themselves. They have similar clinical presentation. Both had psychomotor delay and started walking at the age of 2.5 years. They had speech delay and speak with difficulty. The brothers had tonic-clonic generalized seizure for the first five years of life controlled by treatment.

Individual IV:1 is a 37-year-old man who is distantly related to the proband with moderate-severe DD/ID accompanied with recurrent epileptic seizure developed at infancy and he is currently on Carbimazole. His IQ is < 50 and he is unable to take care of himself and is therefore currently institutionalized. He lacks bladder control and needs assistance with eating food. He understands his surroundings and responds well. His speech is poor and he can only say a few words. His gait is abnormal, characterized with tiptoeing with long steps and poor balance and he needs help with walking. However, he can go up and down stairs.

Individual V:1 is a 8.5-year-old girl who is a cousin of the proband and has a similar clinical presentation as the proband, with seizures, speech impairment, and mild-to-moderate DD/ID. The DNA samples from this patient were not available for genetic analysis.

Table 1 summarizes the clinical features of the seven individuals from the Dutch and Iranian families.

**Table 1:** Comparison of phenotypes in Dutch and Iranian families

	*Family A*		*Family B*				
	II-1	II-2	IV:1	IV:6	IV:3	V-2	V:1
Age at last evaluation (years)	14	8	36	41	34	9	8.5
Gender	M	M	M	M	M	F	F
Ancestry	Dutch	Dutch	Iranian	Iranian	Iranian	Iranian	Iranian
Consanguinity	No	No	Yes	Yes	Yes	Yes	Yes
Weight (kg)	34 (11 years)	20 (5.4 years)	62	60	57	40	32
Height (cm)	143 (11 years)	114.5 (5.4 years)	165	155	160	142	133
Head circumference (cm)	52 (11 years)	51.4 (5.4 years)	Normal	Normal	Normal	Normal	Normal
Cognition and ID	Mild	Mild	Moderate to Severe	Mild	Mild	Mild to Moderate	Mild to Moderate
IQ level	55	68	Below 50	~50	~50	~50	~50
Speech	Dysphasia	Delayed and poor intelligibility	Only a few words	Delayed	Delayed	Incomprehensible but improving	Incomprehensible
Motor function	Delayed	Delayed	Gait abnormality	Delayed	Delayed	Delayed	Delayed
Epilepsy	No	No	Yes	Yes	Yes	Yes	Yes
Muscular abnormality/CK level	No/slightly elevated	No/normal	No/normal	No/normal	No/normal	No/normal	No/Not done
Muscle biopsy	Normal	Not done	Not done	Not done	Not done	Not done	Not done
Vision examination	Normal	Normal	Normal	Normal	Normal	Normal	Normal
Brain imaging	MRI: non-specific white matter changes that resolved later	Not done	Normal CT scan	Normal CT scan	Normal CT scan	MRI: non-specific white matter changes that resolved later	Not done

### Genetic analysis

#### Family A

To identify the genetic defect(s) causing the disease in the affected brothers, exome sequencing was carried out. Under the assumption that homozygous, compound heterozygous, or hemizygous variants are responsible with inclusion of variants present in ≥ 4 reads and present in ≥ 80% of all reads (homozygous) or biallelic in 15–80% of the reads (compound heterozygous) (Table 2), two potential compound heterozygous mutations in the *B3GALNT2* were identified. The compound heterozygous mutation consists of a duplication of two base pairs that leads to a premature stop codon (c.822_823dup, p.Ile276Leufs*26) in exon 7 and a missense mutation (c.988C > T, p.Arg330Cys) in exon 8. Both mutations were verified by Sanger sequencing (Fig. 1c). Segregation analysis in the family showed that the parents carry one of the mutations each and that the healthy brother carries only the missense mutation (Fig. 1a). The *B3GALNT2* mutations were the only variants identified by exome sequencing that could be confirmed and that showed segregation with the phenotype (Table 2). Both mutations are predicted to be pathogenic by SIFT, MutationTaster, and Polyphen2 and have CADD scores > 20 (Table 2). Variant c.988C > T was found in 3/243,112 alleles and c.822_823dup in 57/277,136 alleles in the Genome Aggregation Database (gnomAD), all of them from European populations, but were not present in the dbSNP (build 138), 1000 Genomes Project, the NHLBI Exome Variant Server, The Greater Middle East (GME) Variome Project, or in our in-house databases. The duplication of two base pairs predicts a premature stop codon upstream of the galactosyltransferase domain (Fig. 1d). This could lead to a decrease in the transcript carrying this mutation due to nonsense-mediated RNA decay or to a truncated variant of the B3GALNT2 protein that lacks the functional galactosyltransferase domain. The missense mutation is located in a conserved region within the galactosyltransferase domain (Fig. 1d) and could therefore affect the biochemical activity of B3GALNT2.

**Table 2:** Overview results of WES in Family A

Chr.	Genomic DNA	Ref.	Aberration	Reads	Variation reads^a^	Gene	Messenger RNA	Protein	PhyloP	Confirmed^b^	Segregation^c^	CADD	SIFT	MutationTaster	Polyphen2	Allele frequency in gnomAD
Compound heterozygous variants																
1	235628968		Dup AA	273	57 (21%)	B3GALNT2	c.822_823dup	p.Ile276Leufs*26	3.624	Yes	Yes	-	-	-	-	0.0002057
1	235621948	G	A	100	30 (30%)	B3GALNT2	c.988C > T	p.Arg330Cys	1.708	Yes	Yes	29.3	Deleterious (score: 0)	Disease causing (*p* value: 1)	Probably damaging (score: 1.000)	0.00001234
12	101016068	G	A	.	37 (36%)	GAS2L3	c.664G > A	p.Glu222Lys	5.61	Yes	No^d^	27.6	Deleterious (score: 0.03)	Disease causing (*p* value: 1)	Possibly damaging (score: 0.563)	0.001949
12	101016071	G	A	.	38 (37%)	GAS2L3	c.667G > A	p.Asp223Asn	5.61	Yes	No^d^	24.8	Deleterious (score: 0.02)	Disease causing (*p* value: 1)	Benign (score: 0.392)	0.001956
Variants in known intellectual disability genes																
1	27105725	A	G	.	10 (45%)	ARID1A	c.5336A > G	p.Glu1779Gly	0.69	Yes	No^e^	13.65	Deleterious (score: 0.03)	Disease causing (*p* value: 0.689)	Possibly damaging (score: 0.483)	0

^a^Variation reads: number of variant reads (% of total reads)

^b^Variant confirmed in proband by Sanger sequencing

^c^Segregation of confirmed variant with phenotype in respective family

^d^The variants in GAS2L3 were both inherited from the (healthy) mother

^e^The single confirmed variant in ARID1A was inherited from the (healthy) mother and was not present in the affected brother of the proband

#### Family B

In order to map the chromosomal location of the disease gene in the extended family, we carried out a homozygosity mapping approach using whole genome SNP genotyping data from four affected and two unaffected individuals, assuming that a homozygous mutation is responsible. Homozygosity analysis yielded a single ~ 3.9 Mb homozygosity-by-descent interval defined by flanking heterozygous SNP markers at positions 232,153,793 (rs1475514) and 236,077,778 (rs4660126) (human version GRCh38/hg38) on chromosome 1q42.2-q42.3 (LOD Score: 3.6). Copy number variation (CNV) analysis of microarray SNP genotyping did not detect any potentially pathogenic aberrations in the patients. The region of homozygosity contains 14 protein-coding genes (Table 3) and none of these genes had previously been implicated in autosomal recessive ID (ARID). However, the *B3GALNT2* is contained within this locus, and as mutations in this gene give rise to congenital muscular dystrophy-dystroglycanopathy, together with brain and eye anomalies and ID as prominent features it was further investigated. Exome sequencing data available from individual V:2 identified a novel homozygous missense mutation, c.979G > A in exon 8 of *B3GALNT2* that is predicted to be pathogenic by PolyPhen2, SIFT, PROVEAN, and MutationTaster. The variant results in an asparagine to aspartic acid substitution, p.Asp327Asn (D327N), at a highly conserved residue within the galactosyltransferase domain of the protein [11]. The mutation was validated by Sanger sequencing and co-segregated with the phenotype in the kindred. It was found in 6/244,450 alleles in the GnomAD database but it was not present in the dbSNP (build 138), 1000 Genomes Project, the National Heart, Lung, and Blood Institute (NHLBI) Exome Sequencing Project (ESP), The Greater Middle East (GME) Variome Project, or in our in-house databases of 500 exomes/genomes from unrelated individuals of Middle Eastern/Iranian origin. No other likely candidate variants were identified in exome data and inspection of all rare homozygous variants did not reveal other persuasive candidates in the linked locus.

**Table 3:** The protein-coding genes within the mapped locus uncovered in Family B

Gene name	Protein	Disease association/mode of inheritance
*LYST*	Lysosomal trafficking regulator	AR-Chediak-Higashi syndrome
*GNG4*	Guanine nucleotide-binding protein	-
*B3GALNT2*	Beta-1,3-N-Acetylgalactosaminyltransferase 2	AR-Muscular dystrophy-dystroglycanopathy (congenital with brain and eye anomalies, type A, 11
*TBCE*	Tubulin-specific chaperone E	AR-Encephalopathy, progressive, with amyotrophy and optic atrophy
		AR-Hypoparathyroidism-retardation-dysmorphism syndrome
		AR-Kenny-Caffey syndrome, type 1
*GGPS1*	Geranylgeranyl diphosphate synthase 1	-
*ARID4B*	AT-rich interaction domain-containing protein B	-
*TOMM20*	Translocase of outer mitochondrial membrane 20	-
*IRF2BP2*	Interferon regulatory factor 2-binding protein 2	-
*TARBP1*	TAR RNA-binding protein 1	-
*COA6*	Cytochrome c oxidase assembly factor 6	AR-Cardioencephalomyopathy, fatal infantile, due to cytochrome c oxidase deficiency 4
*KCNK1*	Potassium channel, subfamily K, member 1	-
*MAP3K21*	Mixed-lineage kinase 4	-
*SIPA1L2*	Sipa1-like protein 2	-
*DISC1*	Schizophrenia 9	Susceptibility to schizophrenia

*AR* autosomal recessive

### Complementation assays

To test the potential pathogenicity of the identified mutations and to compare their effect with previously described *B3GALNT2* mutations, complementation experiments were performed. A previously generated *B3GALNT2*-deficient haploid HAP1 cell line (ΔB3GALNT2) was used for complementation with WT and mutant variants of *B3GALNT2* cDNA. To predict the effect on B3GALNT2 enzymatic activity, cells were stained with the IIH6 antibody, recognizing the ligand-binding glyco-epitope on α-DG; subsequently cytometric analysis was performed.

**Fig. 3:**
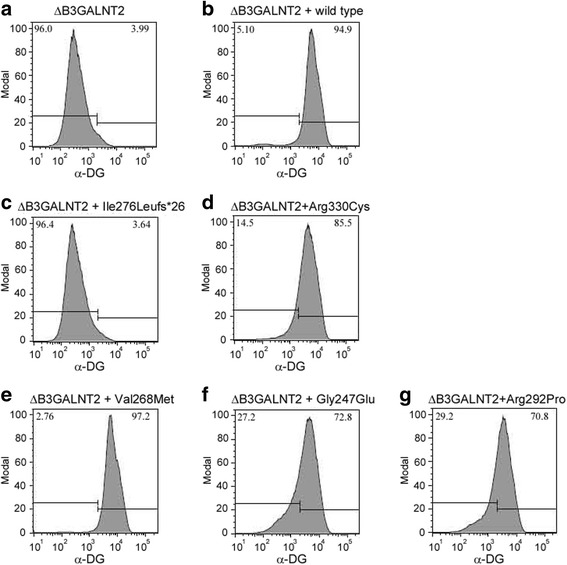
Complementation of *B3GALNT2*-deficient HAP1 cells. **a**–**g** IIH6 FACS analysis of HAP1 *B3GALNT2*-deficient cell lines (ΔB3GALNT2) (**a**) complemented with WT (**b**) and different *B3GALNT2* mutants (**c**–**g**). Percentages of IIH6-positive (*upper right corner*) and IIH6-negative (*upper left corner*) are given. The fluorescent signal of WT cells incubated with only the secondary antibody was used to determine the percentage of IIH6-positive cells

*B3GALNT2*-deficient cells were largely devoid of IIH6 staining (Fig. 3a; 4.0% IIH6-positive cells), emphasizing the importance of B3GALNT2 for O-mannosylation of α-DG. Complementation of the *B3GALTN2*-deficient cells with WT *B3GALNT2* cDNA clearly restored IIH6 staining (Fig. 3b; 94.9% IIH6-positive cells). Complementation with *B3GALNT2* cDNA containing the two base pair deletion (p.Ile276LeuFs*26) failed to restore IIH6 staining (Fig. 3c; 3.6% IIH6-positive cells), indicating that this mutation abolishes B3GALNT2 activity. In contrast, complementation with *B3GALNT2* cDNA containing the missense mutation (p.Arg330Cys) significantly restored IIH6 staining (Fig. 3d; 85.5% IIH6-positive cells as compared to 94.9% for the WT construct), indicating that this mutation only mildly affects B3GALNT2 activity.

Furthermore, complementation with three previously described missense mutations [10] was performed as a comparison to the variants identified here. Remarkably, complementation with *B3GALNT2* cDNA harboring the only homozygous missense mutation (p.Val268Met) that was identified in Stevens et al. did restore IIH6 staining completely (Fig. 3e; 97.2% IIH6-positive cells). Complementation with the two other *B3GALNT2* mutants (p.Gly247Glu and p.Arg292Pro), identified as compound heterozygous variants in one patient, led to partial restoration of IIH6 staining (Fig. 3f, g; 72.8% and 70.8 IIH6-positive cells, respectively), indicating that these variants are not completely non-functional.

## Discussion

In this report, we describe two families affected with ID with and without epilepsy caused by mutations in *B3GALNT2*, a known gene associated with MDDG. Remarkably, the patients present with psychomotor and speech delay, epilepsy, and behavior problems, but no signs of muscular dystrophy and ocular problems, a presentation not previously associated with MDDG syndromes. Although late-onset manifestation of muscular dystrophy in the presented cases, particularly the younger individuals, cannot be excluded, the muscle defects are typically more prevalent than structural brain anomalies and cognitive impairments in previously reported forms of MDDG [18–23]. Two of the patients from these two families had signs of white matter signal intensity changes at a younger age, but these were not seen at a later age.

**Fig. 4:**
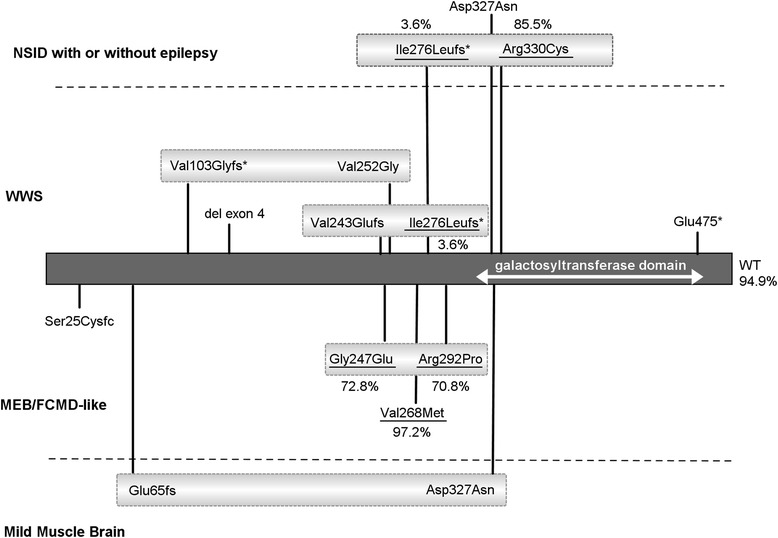
Overview of known *B3GALNT2* mutations, categorized by clinical phenotype. Effect of the underlined mutations was tested by the complementation assay. The relative activity of tested mutations as determined in the complementation assay (Fig. 3) is indicated

Individuals reported with *B3GALNT2* mutations present with severe phenotypes, characterized by cobblestone lissencephaly, congenital muscular dystrophy, and other features indicative of WWS or slightly milder MEB/FCMD-like phenotypes [10]. Recently, a patient was reported with a milder phenotype consisting of psychomotor retardation, ataxia, spasticity, muscle weakness, white matter anomalies, a hypoplastic pons, and subcortical cerebellar cysts [11]. Interestingly the phenotype, caused by a compound heterozygous mutation (p.Asp327Asn/p.Glu65fs*; Fig. 4), includes the p.Asp327Asn variant identified in the Iranian kindred in the homozygous state.

The severity of the mutant *B3GALNT2* phenotype can only be partially explained by the pattern of mutations. Biallelic loss-of-function (LOF) mutations are most commonly seen in association with a WWS phenotype, but in one case also with an MEB/FCMD-like presentation [10] (Fig. 4). Compound heterozygous missense mutations are more often associated with an MEB/FCMD-like disease. Remarkably, the combination of a missense mutation and a frameshift mutation lead to a range of variable phenotypes: WWS; MEB/FCMD-like; the mild muscle-brain phenotype reported by Hedberg et al.; and the mild phenotype without clear muscular involvement reported in this manuscript.

To assess whether the variable phenotypes could be explained by the LOF resulting from the various mutations in *B3GALNT2*, we carried out complementation assays in *B3GALNT2*-deficient HAP1 cells under the hypothesis that severity of phenotype is associated with *B3GALNT2* complete LOF. These experiments revealed that *B3GALNT2* cDNA containing the duplication predicted to cause p.Ile276LeuFs*26 could not restore IIH6-binding, confirming that it represents a LOF allele. Compound heterozygosity of this mutation and another predicted LOF frameshift mutation (p.Val243Glufs*2) is associated with WWS [10]. In the patient described here, the same mutation is found in compound heterozygosity with a p.Arg330Cys substitution. This mutation had a minor effect on B3GALNT2 function, as this *B3GALNT2* variant restored IIH6-binding almost to the level observed for WT HAP1 cells (85.5% IIH6-positive cells vs 94.9% in control). These data suggest a correlation between residual B3GALNT2 activity and phenotype. However, a possible correlation cannot be extrapolated to the activity measurements for all other reported mutations (Fig. 4). Complementation with *B3GALNT2* cDNAs containing either of the compound heterozygous missense mutations associated with a MEB-FCMD-like phenotype (p.Gly247Glu and p.Arg292Pro) showed a partly reduced restoration of the IIH6-binding (72.8% and 70.8%, respectively). In addition, complementation with the p.Val268Met mutation, which was found in the homozygous state in a MEB/FCMD patient, fully restored the IIH6-binding in the *B3GALNT2*-deficient cells (97.2% IIH6-positive cells vs 94.9% in control). A striking observation therefore is that the percentage of IIH6-positive cells is not strictly correlated to the severity of the phenotype. One explanation for this is that there is some redundancy for B3GALNT2 activity, which may lead to a different disease threshold across different cell types and tissues. Possibly, B3GALNT2 has greater redundancy in muscle cells than in neuronal, which would be in contrast to other enzymes involved in O-mannosylation of dystroglycan, for which mutations seem to have a higher threshold in neuronal cells as compared to muscle cells.

It is of note that α-DG skeletal muscle staining using the IIH6 antibody also does not in all cases correlate with the severity of the clinical phenotype, as was shown for patients with a defect in *FKTN* or *FKRP* [24]. However, the severity of the clinical phenotype of *B3GALNT2* patients seems to correlate well with IIH6 muscle staining. In the mildly affected patients described in this study, no significant reduction of IIH6 staining was observed and there was an intact muscular structure (Fig. 1d). In MEB/FCMD-like patients a strongly reduced IIH6 staining and an abnormal muscular structure have been observed [10] and a reduced IIH6 staining in combination with an intact muscle structure was observed for the mild muscle-brain patient [11].

## Conclusions

We show that mutations in *B3GALNT2* can give rise to DD/ID without muscle involvement. This atypical MDDG syndrome phenotype could be classified as a novel form, which is expected to expand due to large scale WES efforts in ID cohorts. This study therefore broadens the spectrum of the MDDG syndromes and highlights the potential for mutations in other MDDG genes to lead to non-syndromic ID as well.

## Abbreviations

CK: Creatine kinase
CNVs: Copy number variations
DD: Developmental delay
DG: Dystroglycan
ESP: Exome Sequencing Project
FCMD: Fukuyama congenital muscular dystrophy
GME: The Greater Middle East
GnomAD: Genome Aggregation Database
ID: Intellectual disability
LGMD: Limb-girdle muscular dystrophy
MDDG: Muscular dystrophy-dystroglycanopathy
MEB: Muscle-eye-brain
MRI: Magnetic resonance imaging
NHLBI: The National Heart, Lung, and Blood Institute
SNP: Single nucleotide polymorphism
WES: Whole exome sequencing
WWS: Walker-Warburg syndrome
